# Association Between Socioeconomic Factors and the Choice of Dentifrice and Fluoride Intake by Children

**DOI:** 10.3390/ijerph8114284

**Published:** 2011-11-10

**Authors:** Carolina Castro Martins, Maria José Oliveira, Isabela Almeida Pordeus, Jaime Aparecido Cury, Saul Martins Paiva

**Affiliations:** 1 Department of Paediatric Dentistry and Orthodontics, School of Dentistry, Federal University of Minas Gerais, Belo Horizonte, Av. Antônio Carlos, Minas Gerais 6627, Brazil; E-Mails: carolcm10@hotmail.com (C.C.M.); isabelapordeus@odonto.ufmg.br (I.A.P.); 2 Department of Paediatric Dentistry and Orthodontics, School of Dentistry, State University of Montes Claros, Campus Darcy Ribeiro, Vila Mauricéia, Montes Claros, Brazil; E-Mail: lagesdeoliveira@gmail.com; 3 Department of Biochemistry, Piracicaba Dental School, University of Campinas (UNICAMP), Av. Limeira 901, Piracicaba, Brazil; E-Mail: jcury@fop.unicamp.br

**Keywords:** children, dentifrice, fluoride, dental fluorosis, socioeconomic factors

## Abstract

It is questionable whether socioeconomic factors influence the choice of marketed children’s dentifrices and whether these products are associated with greater fluoride (F) intake in children. The present cross-sectional study involving 197 children (mean age: 40.98 ± 6.62 months) was carried out in Montes Claros, Brazil. Parents completed a questionnaire on socioeconomic status and the tooth brushing habits of their children. The children brushed their teeth and saliva residues were collected for F analysis. F intake from dentifrice was determined with an ion-specific electrode. Univariate analysis and logistic regression were used to test whether the type of dentifrice (children’s or family) and F dose (<0.05 and ≥0.05 mg F/Kg of body weight/day) were associated with the independent variables (p < 0.05). No differences were found between children’s and family dentifrices regarding daily F intake (0.046 and 0.040 mg F/Kg/day, respectively; p = 0.513). The following were strong predictors for the use of a children’s dentifrice: studying at a private kindergarten (OR: 6.89; p < 0.001); age that the child begun to tooth brush <2 years (OR: 2.93; p = 0.041), and the interaction between the variables “use of the same dentifrice as parents” and “type of tooth brush used” (OR: 27.20; p < 0.001). “The amount of dentifrice used” and “frequency of tooth brushing” (p ≤ 0.004) had a statistically and synergistic effect over the daily F dose. The present study found a social influence over the choice of dentifrice: children with a high socioeconomic status tend to use a children’s dentifrice. The amount of dentifrice used can strongly increase the risk of exposure to higher doses of F, regardless of the type of dentifrice.

## 1. Introduction

Despite the increasing prevalence of dental fluorosis in both developed [1] and developing countries [2], the association between dental fluorosis and fluoride (F) intake by young children is a controversial subject with no strong evidence of the association [3–5]. Among the biological factors, F intake from drinking water and dentifrices are important risk factors for F intake [6] and dental fluorosis [7,8]. Socioeconomic factors have also been suggested as potential risk factors for dental fluorosis [9,10] and F intake level by children [11].

It has been reported that children with a higher socioeconomic status (SES) use a greater amount of dentifrice when tooth brushing and spend more time tooth brushing [12]. Moreover, the brand of the dentifrice seems to be associated with the amount of dentifrice placed on the tooth brush, amount of F ingested and time spent brushing [12]. However, the study [12] only compared SES to brushing habits but there are no data on whether socioeconomic factors influence the choice of a particular type of dentifrice (such as a children’s dentifrice) or whether SES is associated with greater F intake by children from tooth brushing with fluoridated dentifrices.

The aim of the present paper was to determine whether socioeconomic factors and tooth brushing habits are associated with: (1) the purchase of a specific type of dentifrice (children’s or family) and (2) daily F intake by tooth brushing with a fluoridated dentifrice.

## 2. Experimental Section

### 2.1. Ethical Considerations

This cross-sectional study received approval from the Human Research Ethics Committee of Federal University of Minas Gerais (Brazil) under protocol number 278/07.

### 2.2. Subjects

The sample was selected by convenience and comprised all children (n = 208) enrolled at eight kindergartens in the city of Montes Claros, Brazil (0.7, 0.6–0.8 ppm F). Four private and four public kindergartens were selected in order to achieve a balance with regard to socioeconomic status. The kindergartens were randomly drawn from a list compiled by the Municipal Department of Education of Montes Claros. At the time of data collection (2007–2008), Montes Claros had 84 kindergartens (31 public and 53 private). All children enrolled at these kindergartens within the age range at risk for the development of dental fluorosis and whose parents agreed to participate were included.

Eleven children were excluded for the following reasons: nine children used non-fluoridated dentifrice and the parents of two children did not complete the questionnaire. The final sample was comprised of 197 children aged from nine to 48 months (mean age: 40.98 ± 6.62 months).

### 2.3. Pilot Study

Before conducting the main study, a pilot study was conducted with 10 children from a kindergarten not included in the main sample to test the collection method. The parents and the children accepted the protocol well and the parents understood the questionnaire.

### 2.4. Questionnaire

Parents were previously contacted to attend a meeting at the kindergarten, to which they were to bring the tooth brush and dentifrice the child used at home. The parents were informed as to the objectives of study. Those who agreed to participate signed a statement of informed consent and were instructed to respond to a structured questionnaire on their children’s current tooth brushing habits. The questionnaire was self-administered and was administered at the kindergarten by a single investigator (MJO), with the aid of two dental students. The parents were told that there were no wrong or right answers and they should consider their child’s current oral habits.

The questionnaire was structured as follows:

1. Questions on the child’s current tooth brushing habits: if the child used the same dentifrice as their parents or not, frequency of tooth brushing, age at which the child began tooth brushing and who applied the paste to the tooth brush. If the child used the same dentifrice as the parents, the product was considered a family dentifrice. If the child used a different dentifrice from that of the parents, the implication was that the parents bought a special dentifrice for the child or for their several children, a children’s dentifrice. This information was reported by parents and was checked with the brand of dentifrice that the parents took to the kindergarten.
2. Questions related to the socio-demographic characteristics: gender of the child, type of kindergarten (public or private), socioeconomic status (SES) of the family and parental educational level. The SES was evaluated by the ABEPE [13], which is a commonly used questionnaire in Brazil for classifying SES and is based on items possessed by the family, such as the number of radios, televisions, household appliances, cars, *etc*. SES was classified as high, medium or low. Parents provided information on the years of schooling that the head of the family possessed; this variable was dichotomised as 0 to 10 years of schooling and more than 10 years of schooling.

### 2.5. Fluoride Intake from Tooth Brushing with Fluoridated Dentifrices

On the same day, the parents and their children were asked to perform tooth brushing. One at a time, each parent/child pair was led to the bathroom of the kindergarten and instructed to perform tooth brushing with the tooth brush and dentifrice brought from home and to tooth brush in the same way that they usually do at home. The parent and/or child were free to disperse the dentifrice on the tooth brush in their usual manner. The weight of the paste was obtained by weighing the tooth brush on a digital scale (AND SV-200; ±0.01 g) before and after applying the dentifrice. The tooth brushing procedure was observed by the investigator that recorded notes on a structured form. The child performed the tooth brushing in his/her normal way with or without the aid of his/her caregiver. No formal instructions were given regarding brushing technique. If the child requested to rinse his/her mouth, the investigator provided deionised water for rinsing and a plastic cup for collecting the expectorate. The deionised water was prepared at the Biochemistry Laboratory of the Sanitation Company of the state of Minas Gerais (COPASA). The investigator (MJO) was aided by two dental students, who maintained sufficient distance while observing so as not to disturb the normal routine of the procedure.

When the child rinsed her/his mouth and/or spat, the suspension was collected in a plastic cup. Some children rinsed and spat, whereas others did not rinse and still others rinsed and swallowed the water. Distilled deionised water was used to wash the tooth brush vigorously and was also collected in the same plastic cup. The suspension, called the “brushing residue”, was homogenised and the volume was measured. From this material, a 15-mL sample was stored at −4 °C until the determination of the fluoride concentration.

Habits directly observed by the investigator during the tooth brushing were noted on a structured form: type of dentifrice, type of tooth brush, amount of paste placed on tooth brush, whether the child spat the paste out, rinsed the mouth and who brushed the child’s teeth. These directly observed variables were used in data analysis instead of data from the questionnaire in order to minimize information bias [14].

After the tooth brushing session, the children were weighed on a digital scale (Plenna^®^, São Paulo, SP, Brazil) and the dentifrice was collected for subsequent lab analysis. Each child received a kit containing a children’s tooth brush and a fluoridated dentifrice to replace the one supplied by the parents. The children, parents and teachers had not been instructed regarding tooth brushing in order not to alter the habitual technique. On a later date, a meeting was held at the kindergarten to give formal instructions to the parents and teachers regarding recommended oral health care.

### 2.6. Laboratorial Analysis

Analysis was performed at the Biochemistry Laboratory of the Piracicaba School of Dentistry, State University of Campinas, Brazil. The analyst was blinded to the type of dentifrice used by the children. The difference principle was used for the determination of the amount of F ingested during brushing (mg F): the amount of F recovered was subtracted from the amount initially used (weight of dentifrice multiplied by F concentration). The daily dose of F to which the child was exposed was determined by multiplying the average amount of F ingested with the frequency of tooth brushing reported by parents on the questionnaire, divided by the weight of the child (mg of F/Kg of body weight/day). The daily F dose was determined considering total soluble fluoride (TSF), which is the biologically available fluoride that can be absorbed by the body.

The F concentration was determined in the brushing residues (394 samples) in duplicate (788 analyses). Total fluoride (TF) and TSF were determined, totalling 1,576 laboratorial analyses. Each brushing residue was mixed in the plastic cup, from which 2.0 mL was extracted and 0.25 mL of the suspension duplicates were transferred to assay pipettes to determine TF. The remaining suspension was centrifuged (3500 rpm for 10 min) and duplicates of 0.25 mL of the supernatant were transferred to assay pipettes for the TSF analysis (ionic + ionisable). After hydrolysis of the ionisable fluoride (MFP) in 0.25 mL HCl (hydrochloric acid) 2 M for 1 h at 45 °C, the sample was neutralised with 0.5 mL NaOH (sodium hydroxide) 1 M and buffered with 1.0 mL of TISAB II (1.0 M acetate buffer, pH 5.0, containing 1.0 M of NaCl and 0.4% CDTA). The analysis was carried out using a fluoride electrode (Orion model 96-09, Orion Research, Cambridge, MA, USA) coupled to an ion analyser (Orion EA-940), previously calibrated with standards containing 0.125 to 2.0 ppm F under the same conditions as the samples.

### 2.7. Statistical Analysis

The data were analysed using the Statistical Package for Social Sciences (SPSS for Windows, version 18.0, SPSS Inc, Chicago, IL, USA). Descriptive analysis of the variables was carried out to characterise the sample.

Daily F dose was tested for normality using the Kolmogorov-Smirnov test and Levene’s test for homogeneity of variance. As the data had non-normal distribution and variance not homogeneous, the non-parametric Mann-Whitney test for two independent samples (α = 5%) was used to compare means of daily F dose between children who used a children’s dentifrice (n = 104) and those that used a family dentifrice (n = 93). The percentage of F intake during tooth brushing was also compared between the children’s and family dentifrices.

Three dependent variables were defined: type of dentifrice, amount of dentifrice applied during tooth brushing and daily F dose. The type of dentifrice was defined as follows:

1. children’s dentifrices: all with special flavour and special characteristics, containing silica and sodium fluoride (NaF) with TF declared on package varying from 500 to 1100 ppm F (n = 86, 82.7% of the sample of children’s dentifrice), or dentifrices with calcium carbonate (CaCO_3_) and sodium monofluorophosphate (MFP) and TF declared on package varying from 800 to 1200 ppm F (n = 18, 17.3% of the children’s dentifrice) [15].
2. dentifrices for adults: all were mint flavoured, with CaCO_3_ and MFP, TF declared on package varying from 1000 to 1500 ppm F (n = 81, 87% of the adult’s dentifrice), or with silica and NaF and TF declared on package varying from 1,100 to 1,450 ppm F (n = 12, 13% of the adult’s dentifrice) [15].

The amount of dentifrice was dichotomised as <½ the length of the bristles, ½ the length of the bristles and entire length of the bristles. The F dose was dichotomised as <0.05 mg of F/Kg/day and ≥0.05 mg of F/Kg/day. This cut-off point was defined based on the lower threshold limit described by Burt [16]. The threshold limit varies between 0.05–0.07 mg F/Kg body/day [16], but only 30 children (15.2%) surpassed the limit of 0.07 mg F/Kg body/day, and 55 children (27.9%) ingested a dose ≤0.05 mg F/Kg/day. The limit of 0.05 mg F/Kg/day was used because of the bigger sample below this limit.

Univariate analyses (chi-square test and Fisher’s exact test) were performed to test associations between the dependent and independent variables. Significance was set to 5%. SES and parental educational level were also tested for significant associations with the other variables. Only the independent variables with p < 0.25 were selected for the regression analysis model. Using the forward stepwise procedure, two regression models were constructed—one with the type of dentifrice and other with the daily F dose. The variables that remained in the logistic regression model with p < 0.05 were considered risk predictors. The model was also tested for interactions between the predictor variables. For those variables that had more than two categorical options (for example, amount of dentifrice used) dummy variables were created. To be considered interaction α was set at 5%.

Because the variable amount of dentifrice used during tooth brushing had more than two categorical options (<½ of the bristles, ½ of the bristles and all bristles’ extension) it was tested in multivariable logistic regression. The variables that were entered in this model were those with p < 0.25.

## 3. Results

One hundred and four children usually used a children’s dentifrice at home and took this type of dentifrice to the kindergarten (52.8%). Ninety-three children usually used a family dentifrice at home and took this type of dentifrice to the kindergarten (47.2%). Table 1 displays the socioeconomic and tooth brushing variables according to type of dentifrice used at home. The use of a children’s dentifrice was statistically associated with studying at a private kindergarten (48.1%), parents who had more than 10 years of study (64.4%), high and medium SES (35.6–49.0%), use of different dentifrices between child and family (81.5%), an adult brushing the child’s teeth (75.0%) and use of a children’s tooth brush (96.0%) (p < 0.001). Contrarily, the use of family dentifrice was associated to studying in a public kindergarten (82.8%), parents who had 0 to 10 years of study (63.0%), low SES (44.5%), children that used the same dentifrice as the parents (83.5%) and use of tooth brush market for adults (14.0%) (p < 0.001).

Most parents reported a frequency of tooth brushing of twice/day (49.5%), followed by three or more times/day (36.5%) and one time/day (14.0%). No parent reported that their children brushed < 1 time/day. However, there are five missing data due to incomplete answers to this item. The total for this item was 192 answers.

Considering the total of 197, 65 children (33.0%) placed paste on <½ the length of the bristles, 61 (31.0%) placed paste on ½ the length of the bristles and 71 (36.0%) placed pasted on the entire the length of the bristles. Dentifrice placed on the entire length of the bristles was significantly associated with low SES (36.6%), medium SES (40.8%) (p < 0.045) and parental education level of 0 to 10 years of schooling (56.3%) (p < 0.009) (Table 2).

Children who used a children’s dentifrice ingested an estimated daily F dose of 0.046 (±0.079; from <0.000 to 0.785 mg of F/Kg/day) and children who used a family dentifrice ingested a mean dose of 0.040 (±0.047, from <0.000 to 0.251 F/Kg/day). This difference was not statistically significant (Mann-Whitney test, p = 513). Although children’s dentifrices accounted with higher percentage of F intake (53.7 ± 23.9%) during tooth brushing than family dentifrices (44.4 ± 42.6%), this difference was non-significant (p = 0.067). The mean amount of paste used during tooth brushing did not differ between the use of a children’s (0.55 ± 0.37 g) and family dentifrice (0.59 ± 0.36 g) (p = 0.441). Most children (n = 142; 72.1%) ingested a dose of <0.05 mg of F/Kg/day and 55 children (27.9%) ingested a dose of ≥0.05 mg of F/Kg/day.

The following variables were significantly associated with a higher F dose in univariate analysis: parental educational level of less than 10 years of study (61.1%, p = 0.029), higher frequency of tooth brushing (p < 0.001) and greater amount of dentifrice used during tooth brushing (p < 0.001) (Table 3).

Table 4 displays the results of the logistic regression analysis regarding predictive factors for the choice of a children’s dentifrice rather than a family dentifrice (Model 1). The following variables remained in Model 1 and were statistically significant predictors of the use of a children’s dentifrice: studying at a private kindergarten (OR: 6.89; 95% CI: 2.69–17.68; p < 0.001), age that the child begun to tooth brush < 2 years (OR: 2.93; 95% CI: 1.05–8.18; p = 0.041). There was interaction between the variables “use of the same dentifrice as parents” and “type of tooth brush used” (OR: 27.20; 95% CI: 11.42–64.75; p < 0.001).

In Model 2, there was interaction between the dummy variables “frequency of tooth brushing twice/day” and “amount of dentifrice covering the entire length of bristles” (OR: 21.82; 95% CI: 1.07–8.36; p = 0.004) and “frequency of tooth brushing 3 or more times/day” and “amount of dentifrice covering the entire length of bristles” (OR: 6.72; 95% CI: 0.68–7.73; p = 0.005).

No variables were significantly associated with the amount of dentifrice used during tooth brushing in the multivariable logistic regression and the results were not represented in Tables. That means that this model did not find any significant predictor that might explain the use of higher amounts of dentifrice during tooth brushing.

## 4. Discussion

Although not all social demographic variables remained in the final logistic model, there was a tendency of children from lower socioeconomic status to use a children’s dentifrice. In Brazil, studying at a private kindergarten is more common among children with a higher SES. Families that are more apt to buy children’s products (children’s dentifrice and tooth brush) appear to have higher SES and also have greater access to oral health care and information [17]. Moreover, when parents buy a different dentifrice for their children, it is generally a children’s dentifrice. Other studies have demonstrated that when the child uses the same dentifrice as the family, it is usually a family dentifrice [18–20]. These findings underscore the social tendency toward the choice of a children’s dentifrice, which is usually more expensive than family dentifrices. The most used dentifrices in the present study were the children’s dentifrice Tandy^®^ and the family dentifrice Sorriso^®^ [15]. The former costs about US $1.59 and the latter costs about US $0.97. Furthermore, the interaction in model 1 shows that the effect of the dentifrice used by the child being the same as the family or not over the type of dentifrice (market-children or familiar) depends if the child uses an adult’s or infant tooth brush. Moreover, the study found that children <2 years are more prompt to use market-children dentifrice. There is a notion disseminated by the population that infant products must be used by toddlers. And maybe the expensive cost of these products makes parents to change for products market for adults as the child grows.

Although this variable did not remain in the final logistic model, the univariate analysis revealed that a lower parental education level was associated with greater F intake (≥0.05 mg of F/Kg/day). Similar results are reported in a Colombian study, in which children with a lower SES were exposed to significantly higher F dose (0.170 mg F/Kg/day) than those with a high SES (0.045 mg of F/Kg/day) [11]. Dentists should reinforce instructions to families regarding proper oral health practices, especially the use of a small amount of dentifrice during tooth brushing.

A greater amount of dentifrice during tooth brushing by children can increase the risk of exposure to higher F doses and it has been previously confirmed by other studies [20–22]. As tooth brushing frequency is used to calculate the daily F dose, higher frequencies of tooth brushing are expected to increase the risk of F intake. The dummy variable amount of dentifrice covering the entire length of bristles presented interaction with the dummy variables “frequency of tooth brushing twice/day” (p = 0.004) and “3 or more times/day” (p = 0.005). That means that there is a synergistic effect between the amount of the dentifrice used and the frequency of tooth brushing over the dose of F intake. However, it is not prudent to recommend reducing the frequency of tooth brushing, because it could reduce caries prevention. The major concern should be instructing parents to use small amounts of dentifrice during tooth brushing.

Moreover, studies report that children with a lower SES use greater amounts of dentifrice when tooth brushing [11,12]. In the present study, the univariate analysis revealed that children with a lower SES and whose parents had a lower education level were significantly more likely to apply greater amounts of dentifrice on the tooth brush. One may therefore speculate that children from low-income families are at greater risk of exposure to higher F doses. However, the strength of this association should be evaluated further.

The amount of dentifrice was more important than the type of dentifrice for the increased risk of F intake. Studies suggest that children who use a children’s dentifrice apply a greater amount of paste [20,23], which may increase the risk of exposure to higher F doses [12,20,24]. However, no significant association was found in the present study between the type of dentifrice and F intake in univariate analysis (Table 3) or in the comparison of mean F intake (0.046 and 0.040 mg of F/Kg of body weight/day). The mean weight of the paste used during tooth brushing was similar with both dentifrices (0.55 and 0.59 g). This confirms previously published data that children are exposed to high doses of F by tooth brushing with fluoridated dentifrices (0.046 to 0.061 mg F/Kg/day) [18,20], regardless of the type of dentifrice. Some children may even exceed this value (0.130 to 0.106 mg of F/Kg/day) [11]. However, the authors cited [11] considered total fluoride (TF), unlike the present analysis, which considered total soluble fluoride (TSF). TSF was considered to predict the F intake dose due to its bioavailability in the body. TF can be inactivated into insoluble fluoride, which decreases the amount of F absorbed, especially in formulas with calcium carbonate and MFP [15]. That occurs due the reaction of ion F with the calcium from the abrasive forming insoluble CaF_2_ (calcium fluoride) [15]. However, even considering TSF, it is worrisome that, by tooth brushing with the fluoridated dentifrice, approximately 28% of the children in the present study exceeded the threshold limit (≥0.05 mg of F/Kg/day) [16,25]. Furthermore, the children ingested 44 to 54% of the TSF in the dentifrices, which underscores the need to decrease the amount of paste during tooth brushing. Although only 28% of children were exposed to a dose ≥0.05 mg of F/Kg/day, this value may increase if F intake from water and diet is considered. Some dentifrices had high concentration of F (1,500 ppm F) and they would contribute to a higher dose of F intake. However, at least 1,000 ppm of soluble F is considered necessary for a dentifrice to have anticaries effect [26], but the current Brazilian legislation only sets the maximum F concentration at 1,500 ppm F, without mentioning the need of soluble, anticaries active F [2]. Furthermore, this value is related to the declared on the package. Even if the dentifrice has 1,500 ppm TF declared on package, the actual TSF may be lower, as proved by lab analysis of dentifrices with MFP and CaCO_3_ with 1,500 ppm F [15].

The present study has some limitations that should be considered. The questionnaire was answered on the same day as the tooth brushing, which could have influenced the parents. Parents’ reports may lead to information bias [14,27]. The frequency of tooth brushing, which was necessary for the calculation of the daily F dose, was derived from the questionnaire and could be overestimated by parents. The questionnaire was not validated and it was not possible to re-administer the questionnaire to check the reliability of the results due the scholar calendar of the kindergartens. However, the sample was large enough to guarantee comparability of data. There was only one observation of the tooth brushing. Since the parents and the children knew they were being observed, they may have acted differently in front of the investigator than they normally do at home. Maybe some parents would tend to demonstrate a more careful behaviour and could have dispersed more or less dentifrice on the tooth brush or could have helped the child during tooth brushing. Although urinary excretion may be a cheaper and easier way to evaluate F intake by children [6], the present study did not used F urinary excretion. However, this does not invalidate the study since the method of saliva recovered after brushing is well recognized in literature [18,20,28].

The present study confirms that using a smaller amount of dentifrice used during tooth brushing is more important than the specific type of dentifrice. It is important to establish public polices with the aid of the national health authorities and manufacturers of fluoridated dentifrices to create educational campaigns that emphasise the use of small amounts of dentifrice and the importance of supervising children during tooth brushing. Moreover, the results suggest that children from higher socioeconomic status are more likely to buy children’s dentifrice and that children from lower socioeconomic status may be at greater risk of higher doses of F intake. However, this association should be further investigated. Families with a lower socioeconomic status should be closely followed and oral hygiene orientation should be reinforced, as their children may be a greater risk of exposure to higher F doses. Dentists should be particularly concerned with such families, as they have less access to dental services and to oral health information, they may not adopt healthy behaviours. Consequently, due the lack of information, this population may put great amounts of dentifrice on the tooth brush.

## 5. Conclusions

The present study found that socioeconomic factors influence the choice of the dentifrice, as families with a higher socioeconomic status were more likely to buy a children’s dentifrice. Tooth brushing frequency and the amount of fluoridated dentifrice placed on the brush can strongly increase the risk of exposure to higher doses of F doses, regardless of the type of dentifrice.

## Figures and Tables

**Table 1:** Distribution of independent variables according to type of dentifrice (children’s or family) used by children aged < 1 to 4 years old.

Independent variables	Dentifrice			
	Total	Children’s	Family	p-value
	N (%) †	N (%)	N (%)	
Gender				
Male	89 (45.2)	51 (49.0)	38 (40.8)	0.249 *
Female	108 (54.8)	53 (51.0)	55 (59.2)	
Type of kindergarten				
Public	131 (66.5)	54 (51.9)	77 (82.8)	<0.001 *
Private	66 (33.5)	50 (48.1)	16 (17.2)	
Parental educational level (years of schooling)^1^				
0 to 10 years	95 (48.5)	37 (35.6)	58 (63.0)	<0.001 *
More than 10 years of study	101 (51.5)	67 (64.4)	34 (37.0)	
SES^1^				
High	46 (23.5)	37 (35.6)	9 (9.8)	<0.001*
Medium	93 (47.4)	51 (49.0)	42 (45.7)	
Low	57 (29.1)	16 (15.4)	41 (44.5)	
Child uses the same dentifrice as parents^1^				
No	99 (51.0)	84 (81.5)	15 (16.5)	<0.001 *
Yes	95 (49.0)	19 (18.5)	76 (83.5)	
Age that child began tooth brushing^1^				
≥2 years	24 (12.2)	16 (15.4)	8 (8.7)	0.150 *
<2 years	172 (87.8)	88 (84.6)	84 (91.3)	
Frequency of tooth brushing 1				
Once/day	27 (14.0)	14 (14.0)	13 (14.1)	0.892 *
Twice/day	95 (49.5)	48 (48.0)	47 (51.1)	
3 or more times/day	70 (36.5)	38 (38.0)	32 (34.8)	
Who places paste on tooth brush? 1				
Child	25 (12.7)	14 (13.5)	11 (11.8)	0.450 *
Adult	172 (87.3)	90 (86.5)	82 (88.2)	
Who brushes child’s teeth? 2				
Child alone	69 (35.0)	26 (25.0)	43 (46.2)	0.002 *
An adult	128 (65.0)	78 (75.0)	50 (53.8)	
Type of tooth brush used 2				
Children’s	176 (91.2)	96 (96.0)	80 (86.0)	0.013 **
For adults	17 (8.8)	4 (4.0)	13 (14.0)	
Amount of dentifrice used 2				
<½ of bristles	65 (33.0)	37 (35.6)	28 (30.1)	0.717 *
½ of bristles	61 (31.0)	31 (29.8)	30 (32.3)	
Entire length of bristles	71 (36.0)	36 (34.6)	35 (37.6)	
Child spat out paste during brushing 2				
Yes	154 (78.2)	81 (77.9)	73 (78.5)	0.918*
No	43 (21.8)	23 (22.1)	20 (21.5)	
Child rinsed mouth during brushing 2				
Yes	114 (57.8)	65 (62.5)	49 (52.7)	0.164*
No	83 (42.2)	39 (37.5)	44 (47.3)	

* chi-square test,

** Fisher’s exact test;

† Considering total of 100% related to column.

1 Data from questionnaire;

2 Data observed during tooth brushing and noted on file card by investigator.

**Table 2:** Distribution of independent variables according to amount of dentifrice used during tooth brushing of children aged < 1 to 4 years old.

Independent variables	Amount of dentifrice used				

	Total	<½ length of bristles	½ length of ristles	Entire length of bristles	p-value
	N (%) †	N (%)	N (%)	N (%)	
Gender					
Male	89 (45.2)	27 (41.5)	33 (54.1)	29 (40.8)	0.242 *
Female	108 (54.8)	38 (58.5)	28 (45.9)	42 (59.2)	
Type of kindergarten					
Public	131 (66.5)	38 (58.5)	47 (77.0)	46 (64.8)	<0.076 *
Private	66 (33.5)	27 (41.5)	14 (23.0)	25 (35.2)	
Parental educational level (years of schooling) 1					
0 to 10 years	95 (48.5)	21 (32.8)	34 (55.7)	40 (56.3)	<0.009 *
More than 10 years of study	101 (51.5)	43 (67.2)	27 (34.3)	31 (43.7)	
SES 1					
High	46 (23.5)	21 (32.8)	9 (14.8)	16 (22.5)	<0.045 *
Medium	93 (47.4)	31 (48.4)	33 (54.1)	29 (40.8)	
Low	57 (29.1)	12 (18.8)	19 (31.1)	26 (36.6)	
Child uses same dentifrice as parents^1^					
No	99 (51.0)	35 (55.6)	32 (53.3)	32 (45.1)	<0.437 *
Yes	95 (49.0)	28 (44.4)	28 (46.7)	39 (54.9)	
Who places paste on tooth brush? 1					
Child	25 (12.7)	6 (9.2)	9 (14.8)	10 (14.1)	0.574 **
Adult	172 (87.3)	59 (90.8)	52 (85.2)	61 (85.9)	
Type of tooth brush 2					
Children’s	176 (91.2)	56 (90.3)	54 (90.0)	66 (90.3)	0.797**
For adults	17 (8.8)	6 (9.7)	6 (10.0)	5 (7.0)	

* chi-square test,

** Fisher’s exact test;

† Considering total of 100% related to column; Sum may be less than 197 because some parents did not provide SES data.

1 Data from questionnaire;

2 Data observed during tooth brushing and noted on file card by investigator.

**Table 3:** Distribution of independent variables according to F intake dose (<0.05 or ≥0. 05 mg of F/Kg of body weight/day).

Independent variables	Dose (mg of F/Kg/day)			

	Total	<0.05	≥0.05	p-value
	N (%) †	N (%)	N (%)	
Gender				
Male	89 (45.2)	67 (47.2)	22 (40.0)	0.362 *
Female	108 (54.8)	75 (52.8)	33 (60.0)	
Type of kindergarten				
Public	131 (65.5)	92 (64.8)	39 (70.9)	0.411 *
Private	66 (35.5)	50 (35.2)	16 (29.1)	
Parental educational level (years of schooling) 1				
0 to 10 years	95 (48.5)	62 (43.7)	33 (61.1)	0.029 *
More than 10 years of study	101 (51.5)	80 (56.3)	21 (38.9)	
SES^1^				
High	46 (23.5)	37 (26.0)	9 (16.7)	<0.286 *
Medium	93 (47.5)	67 (47.2)	26 (48.2)	
Low	57 (29.0)	38 (26.8)	19 (35.1)	
Type of dentifrice normally used^2^				
Children’s	104 (52.8)	77 (54.2)	27 (49.1)	0.517 *
Family	93 (47.2)	65 (45.8)	28 (50.9)	
Child uses same dentifrice as parents 1				
No	99 (51.0)	71 (50.7)	28 (51.8)	0.887 *
Yes	95 (49.0)	69 (49.3)	26 (48.2)	
Age that child began tooth brushing^1^				
≥2 years	24 (12.2)	20 (14.1)	4 (7.4)	0.233 **
<2 years	171 (87.8)	122 (85.9)	50 (92.6)	
Frequency of tooth brushing 1				
Once/day	27 (14.1)	26 (18.7)	1 (2.0)	<0.001 *
Twice/day	95 (49.5)	69 (49.6)	26 (49.0)	
3 or more times/day	70 (36.4)	44 (31.7)	26 (49.0)	
Who placed paste on tooth brush? 1				
Child	25 (12.7)	18 (12.7)	7 (12.7)	0.580 *
Adult	172 (87.3)	124 (87.3)	48 (87.3)	
Who tooth brushed child’s teeth? 2				
Child alone	69 (35.0)	50 (35.2)	19 (34.6)	0.930 *
An adult	128 (65.0)	92 (64.8)	36 (65.4)	
Type of tooth brush used^2^				
Children’s	176 (91.2)	125 (89.9)	51 (94.4)	0.406 **
For adults	17 (8.8)	14 (10.1)	3 (5.6)	
Amount of dentifrice used 2				
<½ length of bristles	65 (33.1)	56 (39.4)	9 (16.4)	<0.001 *
½ length of bristles	61 (30.9)	47 (33.1)	14 (25.4)	
Entire length of bristles	71 (36.0)	39 (27.5)	32 (58.2)	
Child spat out paste during brushing 2				
Yes	154 (78.2)	110 (77.5)	44 (80.0)	0.697 *
No	43 (21.8)	32 (22.5)	11 (20.0)	
Child rinsed during brushing 2				
Yes	114 (57.9)	83 (58.4)	31 (56.4)	0.790 *
No	83 (42.1)	59 (41.6)	24 (43.6)	

* chi-square test,

** Fisher’s exact test;

† Considering total of 100% related to column; Sum may be less than 197 because some parents did not provide parental education data.

1 Data from questionnaire;

2 Data observed during tooth brushing and noted on file card by investigator.

**Table 4:** Logistic regression analysis regarding type of dentifrice (Model 1) and daily F intake (Model 2).

Predictors	R^2^	OR_adj_	95% CI	p-value
**Model 1**†				
Kindergarten	0.57			
public		1		
private		6.89	2.69–17.68	<0.001
Age that the child begun to tooth brush				
≥2 years		1		
<2 years		2.93	1.05–8.18	0.041
Interaction term				
Child uses same dentifrice as parents (no) ^*^ type of tooth brush (market-children)		1		
		27.20	11.42–64.75	<0.001
**Model 2**‡				
Interaction term	0.28			
Frequency of tooth brushing (twice/day) ^*^ amount of dentifrice used (entire length of bristles)		1		
		21.82	1.07–8.36	0.004
Frequency of tooth brushing (3 or more times/day) ^*^ amount of dentifrice used (entire length of bristles)		1		
		6.72	0.68–7.73	0.005

† Variables with p < 0.25 by chi-square test and Fisher’s exact test incorporated in model (Table 1).

a Variables entered on step 1: type of kindergarten, parental educational level, SES, age that the child begun to tooth brush, who tooth brushes the child’s teeth, the child rinsed during brushing, child uses the same dentifrice as the parents, type of tooth brush used, type of kindergarten ^*^ child uses the same dentifrice as the parents, type of kindergarten ^*^ parental educational level, parental educational level ^*^ child uses the same dentifrice as the parents, parental educational level ^*^ child uses the same dentifrice as the parents

b Variables entered on step 2: gender, parental educational level, SES, age that the child begun to tooth brush, who tooth brushes the child’s teeth, the child rinsed during brushing, type of tooth brush used, type of kindergarten ^*^ child uses the same dentifrice as the parents, type of kindergarten ^*^ type of tooth brush used, type of kindergarten ^*^ parental educational level, parental educational level ^*^ child uses the same dentifrice as the parents, parental educational level ^*^ type of tooth brush used

c Variables entered on step 3: gender, parental educational level, SES, who tooth brushes the child’s teeth, the child rinsed during brushing, child uses the same dentifrice as the parents, type of kindergarten ^*^ type of tooth brush used, type of kindergarten ^*^ parental educational level, parental educational level ^*^ child uses the same dentifrice as the parents, parental educational level ^*^ type of tooth brush used.

‡ Variables with p< 0.25 by chi-square test and Fisher’s exact test incorporated in the model (Table 4): ^a^ Variables entered on step 1: parental educational level, frequency of tooth brushing (twice/day), frequency of tooth brushing (3 or more times/day), amount of dentifrice used (½ of bristles), amount of dentifrice used (entire length of bristles), parental educational level ^*^ frequency of tooth brushing (dummy variables), parental educational level ^*^ amount of dentifrice used (dummy variables), frequency of tooth brushing (dummy variables) ^*^ amount of dentifrice used (dummy variables).
